# Occult metastasis is no burden factor in oral squamous cell carcinoma patients when adhering to a standardized approach in neck dissection

**DOI:** 10.1007/s00784-024-05514-8

**Published:** 2024-01-24

**Authors:** Ann-Kristin Struckmeier, Mayte Buchbender, Tobias Moest, Rainer Lutz, Abbas Agaimy, Marco Kesting

**Affiliations:** 1 Department of Oral and Cranio-Maxillofacial Surgery, Friedrich-Alexander-Universität Erlangen-Nürnberg (FAU), Glückstraße 11, 91054 Erlangen, Germany; 2 Comprehensive Cancer Center Erlangen-European Metropolitan Area of Nuremberg (CCC ER-EMN), Erlangen, Germany; 3 Institute of Pathology, Friedrich-Alexander-Universität Erlangen-Nürnberg (FAU), Erlangen, Germany

**Keywords:** Lymph node metastasis, Neck dissection, Occult metastasis, Oral squamous cell carcinoma

## Abstract

**Objectives:**

Management of the neck in patients with oral squamous cell carcinoma (OSCC) is pivotal to oncologic control and survival. However, there is controversy regarding necessity of neck dissection (ND) in patients with clinically node-negative neck. We aimed to assess risk factors for occult metastasis and to explore whether the presence of occult lymph node metastases (LNMs) has an impact on recurrence and survival.

**Material and methods:**

A retrospective cohort study was performed including patients with primary OSCC who underwent radical tumor resection and ND in a high-volume center adhering to the prevailing German guideline. The ND was performed according to a standardized approach.

**Results:**

Four hundred twenty-one patients with primary surgically treated OSCC were included. The incidence of occult metastasis was 14.49%. A pathological T stage > 1 (multivariate analysis, odds ratio (OR) 3.958, *p* = 0.042) and the presence of extranodal extension in LNMs (multivariate analysis, OR 0.287, *p* = 0.020) were identified as independent risk factors for occult metastasis. When comparing patients with and without occult metastasis, there were no significant differences in terms of progression-free survival (log-rank, *p* = 0.297) and overall survival (log-rank, *p* = 0.320). There were no cases of ipsilateral neck recurrence. One patient developed contralateral neck metastasis; however, he initially presented with a unilateral pT1 pN0 tumor.

**Conclusions:**

Overall, our findings suggest that conducting a standardized approach in ND should be applied in terms of management of the neck in order to maintain survival rates and to prevent neck recurrence in OSCC patients.

Clinical relevance.

None of the risk factors for occult metastasis can be reliably assessed preoperatively. Although elective ND does not guarantee the complete prevention of neck recurrence, it increases the likelihood of either timely removal of micrometastases or strengthens the justification for adjuvant therapy. Consequently, this approach leads to improvements in clinical outcomes.

## Introduction

Oral squamous cell carcinoma (OSCC) accounts for approximately 90% of all malignant tumors within the oral cavity, with a global incidence surpassing 350,000 cases [1, 2].

OSCC is characterized by a high propensity for cervical lymph node metastases (LNMs), affecting approximately 42.6% of the patients [3]. As dissemination to the regional lymph nodes represents the most critical prognostic factor in OSCC patients [4], effective management of the neck is indispensable for oncological control and survival [5]. Despite this, there has been ongoing controversy regarding the optimal approach for patients without clinically detectable LNMs—a debate that has persisted since the 1980s [6].

The prevalence of occult nodal metastasis in clinically node-negative (cN0) necks varies from 7.3 to 36.8% [7, 8], and their progression to clinically evident LNMs is known to be associated with poor oncological outcomes [9]. The proportion of occult metastases naturally depends on the sensitivity of the preoperative clinical examination and the employed diagnostic modalities, e.g., computed tomography or magnetic resonance imaging, as well as the definition of pathological LNMs in imaging [10].

In 1994, Weiss et al. [11] were the first to propose a threshold for recommending ND based on the percentage of occult metastases. They indicated that patients with a risk of occult metastasis greater than 20% have improved regional control, disease-specific survival, and overall survival when undergoing ND [11]. As a general rule of thumb, many clinicians today recommend elective ND when the risk of occult LNM exceeds 15–20% [12, 13].

The primary rationale for advocating routine elective neck dissection (ND) as part of the primary treatment of OSCC patients is the early detection of occult metastasis, enabling adjustments to the adjuvant treatment plan and improving prognosis [14, 15]. However, ND is associated with potential morbidity, such as shoulder pain and dysfunction due to accessory nerve paralysis [13], prompting exploration into less invasive alternatives such as sentinel lymph node biopsy (SNB) or a wait-and-see policy for treatment deintensification.

The objectives of this present study were twofold: first, to identify the risk factors associated with occult metastasis, and second, to investigate whether the presence of occult LNMs has an impact on recurrence along with progression-free survival (PFS) and overall survival (OS) when ND is performed according to a standardized approach.

## Methods

### Study design and participants

The study cohort encompassed patients with primary OSCC, who received treatment including radical tumor resection and ND. The treatment regime adhered to the prevailing German guidelines and was conducted in a high-volume center between January 1, 2013, and May 31, 2023. All treatments were performed according to oncology board meetings’ recommendations.

**Fig. 1:**
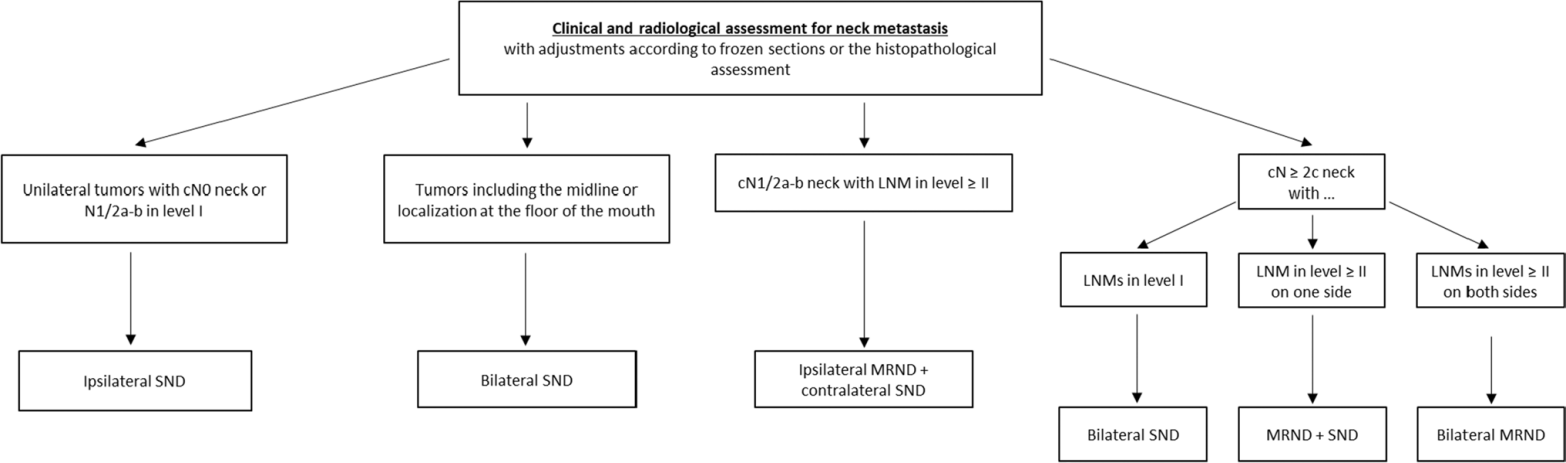
Flow chart for the algorithm of neck dissection in patients with oral squamous cell carcinoma. Abbreviations: cN0 clinically node-negative neck, LNM lymph node metastasis, MRND modified radical neck dissection, SND selective neck dissection

The ND procedure followed a standardized approach as shown in Fig. 1. We consistently performed split-up NDs, as this approach involves dissecting lymph node specimens into packages, allowing for the categorization of LNMs into cervical levels following histopathological analysis [16, 17]. This information empowers clinicians to make decisions about whether to extend the ND to levels IV and V, and to tailor adjuvant radiotherapy [16].

The necessity of adjuvant radiotherapy or radiochemotherapy was determined based on the individual risk factors of each patient, in accordance with the recommendations set forth in the German guideline.

The follow-up schedule was as follows: in the first year, clinical examinations were performed every 6 weeks; in the second year, it was every 3 months; during the third and fourth years, follow-ups were scheduled every 6 months, and in the fifth year, assessments took place after 12 months. Additional computed tomography scans were conducted every 6 months in the first 2 years and every 12 months for the subsequent 3 years.

Exclusion criteria encompassed recurrent OSCC and squamous cell carcinoma of the lip. Additionally, patients who either declined to undergo ND or had a reduced extent of ND due to severe comorbidities were excluded. Moreover, to prevent potential bias stemming from surgery-related short-term mortality, patients who passed away within 30 days following surgery (perioperative death) were excluded from survival analyses. Patients with a follow-up period of less than 30 days were also excluded.

The study’s design and methodologies received approval from the Ethics Committee of the Friedrich-Alexander-University Erlangen-Nuremberg (Ethic votes: 23–185-Br, 23–186-Br). In accordance with national and institutional regulations, written informed consent was not necessary.

The manuscript was prepared according to the STROBE statement.

### Contrast-enhanced computed tomography

Prior to surgery, all patients included in this study underwent preoperative thin-section axial multidetector computed tomography scans for staging. These scans were conducted using an intravenous iodine-based contrast agent to enhance soft tissue differentiation. The assessment of imaging data involved a minimum of two independent physicians from the Department of Radiology. At least one consultant assessed the local extent of the tumor and evaluated the lymph node status.

### Clinicopathological characteristics

Clinicopathological characteristics were obtained from the clinical hospital files. The following parameters were systematically recorded and evaluated: age, sex, tumor localization, TNM classification, depth of invasion (DOI), histological grading, resection margins, presence of perineural, vascular, and lymphovascular invasion, and extranodal extension (ENE). In addition, time point of surgery and time point of last follow-up as well as time point of death were recorded.

The TNM classification was revised during the study period. To ensure the consistency of our results, we restaged patients who were initially classified using the 7th TNM classification. Thereby, all patients were classified according to the 8th TNM classification.

### Statistical analysis

Statistical analysis was performed using the Statistical Package for the Social Sciences 28.0 (SPSS, Chicago, IL, USA).

Correlation analysis was performed using chi-square test.

For evaluating risk factors of occult metastasis, we utilized logistic regression analysis, followed by a multivariate analysis that incorporated factors showing significance in the univariate analysis.

In addition, PFS and OS in patients with and without occult metastasis were estimated using the Kaplan–Meier method. We utilized the log-rank test to compare survival outcomes between the two groups.

PFS was defined as the time elapsed from the day of surgery to locoregional or lymph node/distant metastatic recurrence and was censored on the last day when the patient was alive without any evidence of recurrence. OS was defined as the time from the day of resection to death from any cause and was censored at the last day when the patient was alive.

Figures were also created using SPSS.

Generally, a *p* value < 0.05 was considered statistically significant.

## Results

Our final study cohort compromised 421 patients with primary OSCC treated with radical tumor resection and ND.

Patients’ clinicopathological characteristics are detailed in Table 1.

**Table 1:** Clinicopathological characteristics of the investigated cohort

Characteristics	Number of patients (%)
No. of patients	421
Sex	
Male	260 (61.76)
Female	161 (38.24)
Age	
Median	64
Range	31–93
Pathological tumor stage	
T1	153 (36.34)
T2	108 (25.65)
T3	66 (15.68)
T4a	94 (22.33)
Pathological nodal stage	
N0	278 (66.03)
N1	43 (10.21)
N2a	11 (2.61)
N2b	28 (6.65)
N2c	11 (2.61)
N3b	50 (11.88)
Tumor localization	
Floor of the mouth	150 (35.63)
Tongue	105 (24.94)
Lower jaw	69 (16.39)
Upper jaw	40 (9.50)
Buccal plane	29 (6.89)
Palate	22 (5.23)
Multilocular	6 (1.43)
Grading	
G1	40 (9.50)
G2	216 (51.31)
G3	158 (37.53)
Gx	7 (1.66)
Lymphovascular invasion	
L0	385 (91.45)
L1	34 (8.08)
Lx	2 (0.48)
Vascular invasion	
V0	409 (97.15)
V1	10 (2.38)
Vx	2 (0.48)
Perineural invasion	
Pn0	336 (79.81)
Pn1	83 (19.71)
Pnx	2 (0.48)
Residual tumor	
R0	410 (97.39)
R1	8 (1.90)
Rx	3 (0.71)
Depth of tumor invasion	
≤ 5 mm	175 (41.57)
6–10 mm	112 (26.60)
> 10 mm	101 (23.99)
DOIx	33 (7.84)
Extranodal extension (% of LNMs)	
ENE(-)	81 (56.64)
ENE( +)	58 (40.56)
ENEx	4 (2.80)

Abbreviation: *LNM* lymph node metastasis

The median age of the patient cohort was 64 years, with a range between 31 and 93 years. The included patients were predominantly male (260/421, 61.76%) and the majority of the tumors were localized either at the floor of the mouth (150/421, 35.63%) or at the tongue (105/421, 24.94%).

Further analysis revealed that 175 (41.57%) of the tumors exhibited a DOI of 5 mm or less, 112 (26.60%) fell within the 6–10 mm range, and 101 (23.99%) had a DOI exceeding 10 mm.

Overall, the incidence of LNMs was 33.97%, encompassing 143 out of the 421 patients. Of these patients, 58 (constituting 40.56%) presented LNMs accompanied with ENE. Occult metastases were identified in 61 out of the 421 patients, equating to a rate of 14.49% (see Table 4). 9.26% (39/421) of OSCC patients were falsely diagnosed with nodal disease preoperatively (false positive rate).

**Fig. 2:**
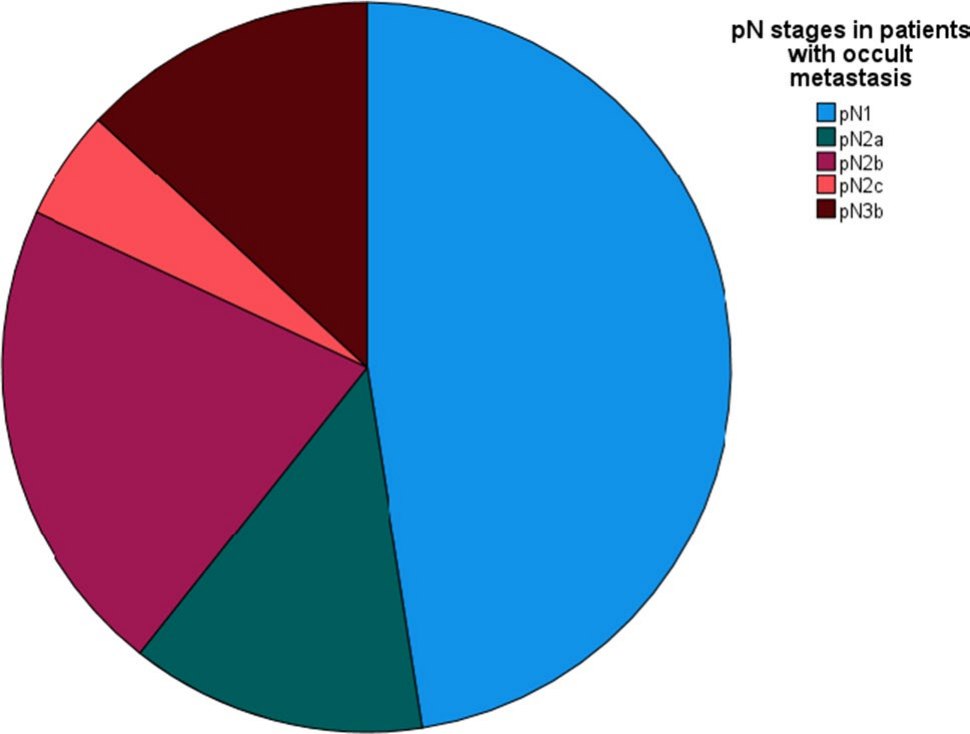
Distribution of pathological nodal staging among patients with occult metastasis

Approximately half of the patients (29/61) with occult metastasis exhibited a single LNM, classifying them as N1 (representing 47.54%). An additional 13.11% of patients had a single LNM with ENE (8 out of 61 patients). Moreover, 21.31% of patients had multiple LNMs ipsilateral without ENE, leading to their classification as N2b (13 out of 61 patients). Furthermore, 4.92% (3 out of 61) and 13.11% (8 out of 61) of patients were categorized as N2c because of bilateral or contralateral metastasis, and N3b due to the presence of ENE along with more than one LNM. The distribution of N staging among patients with occult metastasis is depicted in Fig. 2.

In this study, 60.81% (256 out of 421) of the patients received adjuvant treatment, such as brachytherapy, radiotherapy, or radiochemotherapy. However, 29 patients (6.89%) chose to either decline adjuvant therapy or did not complete it, although it was recommended.

### Correlation between clinicopathological characteristics and occult metastasis

Several factors were significantly associated with the presence of occult metastasis. These factors included pathological T stage (chi-square, *p* < 0.001), pathological N stage (chi-square, *p* < 0.001), presence of ENE (chi-square, *p* < 0.001), lymphovascular (chi-square, *p* = 0.010) and perineural invasion (chi-square, *p* = 0.002), grading (chi-square, *p* = 0.015), and DOI (chi-square, *p* < 0.001). The results of the correlation analysis are depicted in Table 2.

**Table 2:** Prevalence of occult metastases according to clinicopathological characteristics

		No occult metastasis (%)	Occult metastasis (%)	Correlation (chi-square)
Sex	**Male**	220 (84.62)	40 (15.38)	0.507
	**Female**	140 (86.96)	21 (13.04)	
Age	** < 65 years**	189 (82.89)	39 (17.11)	0.126
	** ≥ 65 years**	171 (88.60)	22 (11.40)	
Pathological tumor stage	**T1**	142 (92.81)	11 (7.19)	< 0.001*
	**T2**	85 (78.70)	23 (21.30)	
	**T3**	47 (71.21)	19 (28.79)	
	**T4a**	86 (91.49)	8 (8.51)	
Pathological nodal stage	**N0**	278 (100.00)	0 (0.00)	< 0.001*
	**N1**	14 (32.56)	29 (67.44)	
	**N2a**	3 (27.27)	8 (72.73)	
	**N2b**	15 (53.57)	13 (46.43)	
	**N2c**	8 (72.73)	3 (27.27)	
	**N3b**	42 (84.00)	8 (16.00)	
Grading	**G1**	39 (97.50)	1 (2.50)	0.015*
	**G2**	188 (87.04)	28 (12.96)	
	**G3**	127 (80.38)	31 (19.62)	
Lymphovascular invasion	**L0**	334 (86.75)	51 (13.25)	0.010*
	**L1**	24 (70.59)	10 (29.41)	
Vascular invasion	**V0**	349 (85.33)	60 (14.67)	0.679
	**V1**	9 (90.00)	1 (10.00)	
Perineural invasion	**Pn0**	296 (88.10)	40 (11.90)	0.002*
	**Pn1**	62 (74.70)	21 (25.30)	
Depth of invasion	** ≤ 5 mm**	160 (92.43)	15 (8.57)	0.004*
	**6**–**10 mm**	89 (79.46)	23 (20.54)	
	** > 11 mm**	80 (79.21)	21 (20.79)	
Extranodal extension	**ENE( +)**	45 (76.27)	14 (23.73)	< 0.001*
	**ENE(-)**	39 (46.99)	44 (53.01)	

A *p* value < 0.05 was considered statistically significant. Statistically significant differences are marked with an asterisk

### Risk factors for occult metastasis

Furthermore, we conducted univariate and multivariate analyses to identify risk factors for occult metastasis.

In the univariate logistic regression analysis, several factors emerged as prognostic indicators for the presence of occult metastasis. Specifically, pathological T stage greater than 1 (odds ratio (OR): 2.961, 95% CI 1.491–5.880, *p* = 0.002), pathological N stage greater than 1 (OR 4.738, 95% CI 4.738, *p* < 0.001), and the presence of perineural invasion (OR 2.506, 95% CI 1.383–4.544, *p* = 0.002) were identified as significant factors. Moreover, patients with lymph node metastases exhibiting ENE were found to be less likely to have occult LNMs (OR 0.276, 95% CI 0.132–0.577, *p* < 0.001).

Subsequently, the multivariate analysis confirmed that pathological T stage greater than 1 (OR 3.958, 95% CI 1.048–14.944, *p* = 0.042) and the presence of ENE (OR 0.287, 95% CI 0.118–0.698, *p* = 0.020) are independent risk factors for occult metastasis.

Detailed results of both the univariate and multivariate analyses can be found in Table 3.

**Table 3:** Univariate and multivariate analysis of the risk factors for occult metastasis

	Univariate analysis			Multivariate analysis		
	OR	95% CI	*p* value	OR	95% CI	*p* value
Age: < 65 vs. ≥ 65 years	0.623	0.355–1.094	0.097			
Sex: male vs. female	0.825	0.467–1.457	0.507			
Pathological T stage: T1 vs. higher	2.961	1.491–5.880	0.002*	3.958	1.048–14.944	0.042*
Pathological nodal stage: N1 vs. higher	4.738	2.686–8.358	< 0.001*	2.077	0.861–5.007	0.104
Grading: G1 vs. higher	7.305	0.984–54.208	0.052			
Lymphovascular invasion: L0 vs. L1	2.729	1.233–6.039	0.10			
Vascular invasion: V0 vs. V1	0.646	0.080–5.194	0.679			
Perineural invasion: Pn0 vs. Pn1	2.506	1.383–4.544	0.002*	0.716	0.324–1.581	0.408
Depth of invasion: < 5 mm vs. ≥ 5 mm	2.777	1.487–5.186	0.001*	0.686	0.176–2.673	0.587
Extranodal extension: ENE(-) vs. ENE( +)	0.276	0.132–0.577	< 0.001*	0.287	0.118–0.698	0.020*

A *p* value < 0.05 was considered statistically significant. Statistically significant differences are marked with an asterisk

Abbreviations: *CI* confidence interval, *DOI* depth of invasion, *ENE* extranodal extension, *G* grading, *L* lymphovascular invasion, *N stage* nodal stage, *OR* odds radio, *p* perineural invasion, *T stage* tumor stage, *v* vascular invasion

### Nodal metastases and their occult percentage depending on tumor subsite and pathological T stage

In the next step, we analyzed the prevalence of occult metastasis while considering tumor localization and pathological T stage. For all T1 tumors, regardless of their localization, the frequency of occult metastasis was approximately 7%.

Conversely, among T2 tumors, the frequency of occult metastasis exceeded 20% for all tumor subsites, with the exception of the hard palate (1 out of 8, 12.50%).

Data regarding the incidence of occult metastasis, both in total and based on tumor subsite and T stage, can be found in Table 4.

**Table 4:** Incidence of occult metastases depending on tumor localization and pathological T stage

	Occult metastasis (%)						
T stage	Overall	Floor of the mouth	Tongue	Lower jaw	Upper jaw	Hard palate	Buccal plane
T1	11 (7.19)	4 (7.27)	3 (6.67)	1 (7.14)	0 (0.00)	2 (11.76)	1 (8.33)
T2	23 (21.30)	7 (20.59)	8 (20.51)	4 (25.00)	1 (20.00)	1 (12.50)	1 (20.00)
T3	19 (28.79)	9 (32.14)	7 (35.00)	1 (11.11)	1 (25.00)	1 (50.00)	0 (0.00)
T4a	8 (8.51)	4 (12.12)	1 (100.00)	1 (3.33)	1 (4.55)	0 (0.00)	1 (3.33)
Overall	61 (14.49)	24 (16.00)	19 (18.10)	7 (10.14)	3 (7.50)	4 (13.79)	3 (13.64)

A *p* value < 0.05 was considered statistically significant. Statistically significant differences are marked with an asterisk

Abbreviation: *T stage* tumor stage

### Association between depth of invasion and the occurrence of occult metastasis

The percentage of occult metastasis in patients with tumors exhibiting a DOI of 3, 4, and 5 mm was 22.22% (6/27), 8.11% (3/37), and 19.35% (6/31), respectively. Overall, the percentage of occult metastasis was 8.57% (15/175) in tumors ≤ 5 mm. Furthermore, the frequency of occult metastasis was 20.54% (23/112) and 20.79% (21/101) in tumors with DOIs between 6–10 and > 11 mm.

Data regarding the incidence of occult metastasis depending on DOI can be found in Table 5.

**Table 5:** Incidence of occult metastasis depending on depth of invasion

Depth of invasion (mm)	Occult metastasis (%)
≤ 2	0 (0.00)
3	6 (22.22)
4	3 (8.11)
5	6 (19.35)

### Survival analysis

We conducted a survival analysis to compare the survival outcomes between patients with occult metastasis and those without. In our patient cohort, there were no significant differences in terms of PFS (log-rank, *p* = 0.297) and OS (log-rank, *p* = 0.320) between these two groups.

**Fig. 3:**
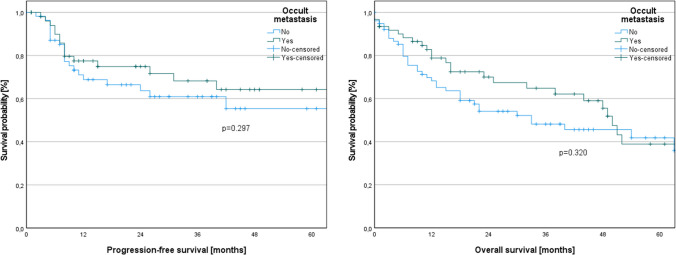
Kaplan–Meier curves of progression-free survival and overall survival depending on the presence of occult metastasis. There were no significant differences in terms of progression-free survival (log-rank, *p* = 0.297) and overall survival (log-rank, *p* = 0.320) between these two groups

The corresponding Kaplan–Meier curves are displayed in Fig. 3.

### Analysis of recurrence

Among our study cohort, 37 patients experienced local recurrence (8.79%) and 6 patients presented with simultaneous occurrences of a local recurrence along with cervical metastases (1.42%). Notably, there were no instances of isolated ipsilateral cervical metastases during the follow-up. One patient, who initially exhibited a unilateral pT1 pN0 tumor, developed contralateral neck metastasis approximately 1 year after the primary surgery (0.24%). Additionally, 15 patients (3.56%) developed distant metastases without any concomitant local or regional recurrence.

## Discussion

OSCC is characterized by a high propensity for cervical LNMs, affecting approximately 42.6% of patients [3]. As dissemination to the regional lymph nodes represents the most critical prognostic factor [4], effective management of the neck is indispensable for oncological control and survival [5].

Nonetheless, there remains an ongoing debate regarding the optimal approach for patients without clinical evidence of metastasis. This debate centers on whether to adopt a wait-and-see policy, utilize less invasive techniques such as SNB, or choose elective ND, particularly in cases involving early-stage tumors.

The objectives of the present study were to evaluate the risk factors associated with occult metastasis and to investigate whether the presence of occult LNMs has an impact on recurrence and survival when ND is conducted adhering to a standardized approach.

Four hundred twenty-one patients with primary surgically treated OSCC were included in the study. Our patient cohort exhibited an incidence of cervical metastasis of 33.97% with 14.49% of patients exhibiting occult metastasis.

Previously, factors such as perineural invasion, lymphovascular invasion, DOI [18], tumor budding [19], and tumor thickness [20] have all been described to be associated with heightened rates of occult metastasis in OSCC patients [21, 22].

When evaluating predictors of occult metastasis among our study cohort, univariate logistic regression revealed pathological T stage > 1 (logistic regression, *p* = 0.002), pathological N stage > 1 (logistic regression, *p* < 0.001), and presence of perineural invasion (logistic regression, *p* = 0.002) as prognostic factors regarding the presence of occult metastasis. In addition, patients with ENE were less likely to have occult metastasis (logistic regression, *p* < 0.001). Multivariate analysis confirmed pathological T stage > 1 (multivariate analysis, *p* = 0.042) and presence of ENE (multivariate analysis, *p* = 0.020) as independent factors.

However, the disadvantage of all these factors, as mentioned above and as evident in our analysis, is that none of them can be reliably assessed before the radical tumor resection and removal of LNM, thereby significantly diminishing their clinical utility in evaluating the preoperative lymph node status.

Analyzing the prevalence of occult metastasis while considering tumor localization and T stage, it was found that the frequency remained consistently at approximately 7% for T1 tumors, regardless of the tumor’s localization within the oral cavity. Conversely, for T2 tumors, the frequency of occult metastasis exceeded 20%, except for those located at the hard palate. Nevertheless, the relatively small number of patients with carcinomas of the hard palate who were included in this study could account for this particular result. In summary, our findings suggest that all tumor localizations within the oral cavity carry a similar risk of presenting occult metastasis.

In similarity with our results, Yang et al. [23] reported an incidence of occult metastasis at 10.9% in patients with T1 tumors localized at the tongue. However, they observed a substantially higher incidence of 28.6% for those with T2 tumors [23]. Furthermore, Hutchison et al. [24] reported even higher rates, with 20.8% for T1 tumors and 36.0% for T2 tumors.

Regarding the T stage, it is noteworthy that the TNM classification underwent a substantial revision in 2017, incorporating changes that include the consideration of DOI and ENE as criteria for determining the T and N stages in patients with OSCC. As a result, conclusions drawn from the 7th TNM classification (which is relevant to both of the aforementioned studies [23, 24]) or studies involving a heterogeneous patient population may no longer provide dependable insights when assessing the occurrence of occult metastasis across various T stages.

Within our patient cohort, the risk of occult metastasis was at 22.22%, 8.11%, and 19.35% for tumors with a DOI of 3, 4, and 5 mm, respectively. In a broader context, the overall rate of occult metastasis was 8.57% for tumors with a DOI of 5 mm or less. Moreover, the incidence of occult metastasis was notably higher at 20.54% and 20.79% for tumors with DOIs falling in the range of 6–10 mm and exceeding 11 mm, respectively.

de Matos et al. [25] reported that among patients with pathological DOIs of 10 mm or less, the proportion of occult LNMs was 15.3%, whereas for those with pathological DOIs exceeding 10 mm, the proportion was substantially higher at 54.2%. They also determined a cutoff value of 10 mm for DOI as a prognostic factor [25].

Kane et al. [26] emphasized that DOI is the most significant histopathological predictor of occult metastasis in OSCC patients and that tumors with a DOI of 5 mm or more are at a heightened risk of nodal metastasis. Considering these findings, some surgeons prefer to use SNB or opt for a wait-and-see approach instead of conducting elective ND when the DOI falls within the 2 to 4 mm range in order to minimize surgical morbidity [27]. Nevertheless, there remains an ongoing debate about the precise DOI threshold that should trigger the decision to opt for elective ND.

The National Comprehensive Cancer Network guideline recommends elective ND when the DOI exceeds 3 mm [28]. On the contrary, Schilling et al. [29] even propose the potential utility of SNB for DOIs up to 10 mm.

It is essential to emphasize that the body of data concerning SNB remains somewhat limited, particularly within the context of prospective studies. A significant limitation in many previous investigations regarding SNB has been the inconsistent histological examination applied to non-sentinel lymph nodes. Furthermore, the reported success rates exhibit significant variation. For instance, Guerlain et al. reported a success rate of 93%, whereas in other studies, the reported success rates frequently fall well below 80% [30–32]. In addition, SNB is not suitable for all tumors, particularly those located at the floor of the mouth [33]. This limitation arises from the challenges associated with the “shine-through phenomenon” [34].

ENE holds substantial prognostic significance and serves as a pivotal factor in the risk assessment of OSCC patients, including considerations for adjuvant therapy [35]. In our analysis, patients with LNMs with ENE were less likely to present with occult metastasis (multivariate analysis, *p* = 0.020). However, the preoperative assessment of ENE is limited in terms of sensitivity and thereby making it impractical to make reliable determinations about its presence before surgery [36, 37].

The association of perineural invasion (*p* = 0.002) with occult LNMs in univariate analysis suggests that elective ND should be considered when this histopathological feature is present, even in the absence of other high-risk histopathologic features. Nonetheless, the perineural invasion did not yield statistical significance in the multivariate analysis (*p* = 0.408).

Occult metastasis was previously described as a burden factor in OSCC patients [38, 39]. However, within our patient cohort, no significant differences were found between patients with and without occult metastasis in terms of PFS (log-rank, *p* = 0.297) and OS (log-rank, *p* = 0.320).

Haidari et al. [38] conducted a study examining the impact of occult metastasis on survival and found that the presence of occult metastasis had a negative influence on PFS in OSCC patients (hazard ratio = 2.33). However, in contrast to our results, they reported a much lower prevalence of occult metastasis of 7.08% with a significantly higher false positive rate of 23.45% [38]. Broglie et al. [39] found similar results with occult metastasis resulting in decreased overall survival and disease-free survival. However, they employed SNB, potentially resulting in missed cases of LNMs [39].

Several studies have examined the disparity in survival outcomes and rates of recurrence between elective and therapeutic ND following a wait-and-see strategy. The results by Fasunla et al. [12] and D’Cruz et al. [13] revealed that elective ND was associated with significantly higher rates of OS and disease-free survival. Furthermore, D’Cruz et al. [13] reported that a greater proportion of patients received adjuvant radiotherapy based on nodal indications following elective neck dissection. In contrast, findings by Liu et al. [40, 41] suggested that adopting a wait-and-see policy does not appear to compromise survival.

This contradicts the findings of several trials indicating that a substantial proportion of patients with early OSCC who undergo a wait-and-see policy will develop neck recurrences, many of which will present with advanced stages and unfavorable prognostic factors like ENE [42]. For example, D’Cruz documented a neck recurrence rate of 45% [26], while Nieuwenhuis et al. [44] and Flach et al. [43] reported lymph node recurrence rates of 21% and 28%, respectively. Ho et al. [45] documented that the survival rates for these patients with recurrences were merely 30%. The elevated recurrence rates could be attributed to the presence of micrometastases (metastases smaller than 2 mm) preoperatively, which are challenging to assess due to the limitations imposed by the slice thickness in image-based techniques.

In this context, it is worth noting that in our patient cohort, there were no instances of ipsilateral LNMs observed during the follow-up period. One patient experienced contralateral neck metastasis approximately 1 year after the initial surgery (0.2%); notably, this patient initially presented with a unilateral pT1 pN0 tumor.

In contemporary medical practice, the pursuit of less invasive techniques to preserve the postoperative quality of life for patients is undoubtedly a priority. To avoid potential morbidity, some surgeons decline ND in the early stages of OSCC.

Certain anatomical structures, including the ramus marginalis n. facialis, accessory nerve, hypoglossal nerve, vagus nerve, and lingualis nerve, are susceptible to injury and potential morbidity during ND [46]. Nevertheless, elective, supraomohyoid ND typically allows for the preservation of these structures in nearly all cases, with accidental injuries being a rare occurrence [47]. On the contrary, these structures can also be at risk during SNB. Schiefke et al. [48] even described similar limitations regarding the quality of life between patients treated with SND and elective ND.

In situations involving the radical resection of advanced LNMs with ENE due to recurrence of the neck, preserving these structures is often not feasible, leading to inevitable compromises in the patient’s quality of life [47]. As described before, the likelihood of advanced LNMs is higher when a wait-and-see approach is chosen over elective ND in cN0 neck patients [42]. As a result, elective ND usually has a lower level of invasiveness compared to therapeutic ND and is thereby associated with less impairment of quality of life.

### Limitations

The main limitations of this study are the sample size and the retrospective methodology. Although previous studies have examined rates of occult LNMs in OSCC, most had smaller sample sizes and contained heterogeneous data.

## Conclusions

In conclusion, our findings suggest that conducting a standardized approach in ND should be applied in terms of the management of the neck in order to maintain survival rates and to prevent neck recurrence in OSCC patients. None of the risk factors for occult metastasis can be reliably assessed preoperatively. While elective ND may not guarantee the prevention of late metastases, it does enhance the likelihood of either timely removal of micrometastases or strengthens the justification for adjuvant therapy, ultimately leading to improved clinical outcomes.

In contemporary medical practice, the pursuit of less invasive techniques to preserve the postoperative quality of life for patients is undoubtedly a priority. However, it is currently inadvisable to select approaches like sentinel SNB or a wait-and-see policy solely with the intention of minimizing immediate morbidity. Such choices may heighten the risk of developing advanced lymph node disease at a later stage, which, in turn, could result in a less favorable prognosis and increased morbidity when therapeutic ND becomes necessary.

## Data Availability

The data that support the findings of this study are available from the corresponding author upon reasonable request.

## Abbreviations

CI: Confidence interval
cN0: Clinically node negative
DOI: Depth of invasion
ENE: Extranodal extension
OSCC: Oral squamous cell carcinoma
LNM: Lymph node metastasis
N stage: Nodal stage
OR: Odds ratio
OS: Overall survival
PFS: Progression-free survival
SNB: Sentinel lymph node biopsy
TNM: Tumor, nodus, metastasis
T stage: Tumor stage
